# Does the time of the day affect multiple trauma care in hospitals? A retrospective analysis of data from the TraumaRegister DGU®

**DOI:** 10.1186/s12873-021-00525-0

**Published:** 2021-11-13

**Authors:** Stefanie Fitschen-Oestern, Sebastian Lippross, Rolf Lefering, Tim Klüter, Matthias Weuster, Georg Maximilian Franke, Nora Kirsten, Michael Müller, Ove Schröder, Andreas Seekamp, TraumaRegister DGU

**Affiliations:** 1 Department of Trauma Surgery, University Medical Center of Schleswig-Holstein, University of Kiel, Arnold-Heller Straße 7, 24105 Kiel, Germany; 2 Institute for Research in Operative Medicine (IFOM), University Witten/Herdecke, Cologne, Germany; 3 Committee on Emergency Medicine, Intensive Care and Trauma Management (Sektion NIS) of the German Trauma Society (DGU), Berlin, Germany

**Keywords:** Multiple trauma, TraumaRegister DGU®, Day shift, Night shift, Admission

## Abstract

**Background:**

Optimal multiple trauma care should be continuously provided during the day and night. Several studies have demonstrated worse outcomes and higher mortality in patients admitted at night. This study involved the analysis of a population of multiple trauma patients admitted at night and a comparison of various indicators of the quality of care at different admission times.

**Methods:**

Data from 58,939 multiple trauma patients from 2007 to 2017 were analyzed retrospectively. All data were obtained from TraumaRegister DGU®. Patients were grouped by the time of their admission to the trauma center (6.00 am–11.59 am (morning), 12.00 pm–5.59 pm (afternoon), 6.00 pm–11.59 pm (evening), 0.00 am–5.59 am (night)). Incidences, patient demographics, injury patterns, trauma center levels and trauma care times and outcomes were evaluated.

**Results:**

Fewer patients were admitted during the night (6.00 pm–11.59 pm: 18.8% of the patients, 0.00–5.59 am: 4.6% of the patients) than during the day. Patients who arrived between 0.00 am–5.59 am were younger (49.4 ± 22.8 years) and had a higher injury severity score (ISS) (21.4 ± 11.5) and lower Glasgow Coma Scale (GCS) score (11.6 ± 4.4) than those admitted during the day (12.00 pm–05.59 pm; age: 55.3 ± 21.6 years, ISS: 20.6 ± 11.4, GCS: 12.6 ± 4.0). Time in the trauma department and time to an emergency operation were only marginally different. Time to imaging was slightly prolonged during the night (0.00 am–5.59 am: X-ray 16.2 ± 19.8 min; CT scan 24.3 ± 18.1 min versus 12.00 pm- 5.59 pm: X-ray 15.4 ± 19.7 min; CT scan 22.5 ± 17.8 min), but the delay did not affect the outcome. The outcome was also not affected by level of the trauma center. There was no relevant difference in the Revised Injury Severity Classification II (RISC II) score or mortality rate between patients admitted during the day and at night. There were no differences in RISC II scores or mortality rates according to time period. Admission at night was not a predictor of a higher mortality rate.

**Conclusion:**

The patient population and injury severity vary between the day and night with regard to age, injury pattern and trauma mechanism. Despite the differences in these factors, arrival at night did not have a negative effect on the outcome.

## Background

Multiple trauma is one of the leading causes of death and disability worldwide [1, 2], and severe injuries must be diagnosed and treated 24 h per day.

Many multiple trauma patients arrive at the trauma center during the evening and night [3, 4]. In particular, presentation at night has been identified as a risk factor for poor outcomes in critically ill patients needing a prompt diagnosis and the timely implementation of interventions [5, 6]. Emergency care during the night can be complicated by medical errors [7], increased complications [8] and less frequent use of aggressive interventions [9]. Decreases in the cognitive and physical performance of medical staff during the night [10–12] might cause delays and malpractice. Additionally, staff density is reduced during the night. Medical diagnostics such as X-rays and CT scans take longer because there are fewer radiology staff members available.

The quality of multiple trauma care in the first 24 h is determined by the timeliness of making a diagnosis and initiating interventions and the time lost by waiting [13]. Specific trauma care in the emergency room, the rapid performance of diagnostic procedures without delay and direct transfer to intensive care or the operating room determine the length of hospital stay and outcomes [14]. Previous studies in emergency care have shown a higher mortality rate and longer hospital stay after patient admission during the night shift [15, 16]. In this study, we evaluated novel data for different quality indicators of multiple trauma care during the night.

This study evaluated whether the time of day at which a multiple trauma patient arrived at the trauma center affected the timing of medical care during the first 24 h and the mortality rate. We used TraumaRegister DGU® [17] to evaluate the current demographic data and the timing of medical interventions in multiple trauma patients admitted during the day and at night. The collection of data from the TR-DGU enabled the evaluation of multiple trauma patients treated in different trauma centers. Data on transport methods, care entities and patient populations were objectively compared.

## Methods

### TraumaRegister DGU®

The TraumaRegister DGU® is a multicenter database with the aim of pseudonymized and standardized documentation of severely injured patients. The database of the German Trauma Society (Deutsche Gesellschaft für Unfallchirurgie, DGU) was founded in 1993. Data included in the TraumaRegister DGU® are collected prospectively. Patients with an admission to the hospital via trauma center with subsequent admission to ICU/ICM (intensive care unit/intermediate care medicine) or death after arrival at the hospital and before admission to the ICU were included. Information on demographics; the injury pattern; comorbidities; pre- and in-hospital management; the clinical course during treatment in the ICU (intensive care unit); relevant laboratory findings, including data on transfusions; and outcomes are documented in the register. The AUC - Academy for Trauma Surgery (AUC - Akademie der Unfallchirurgie GmbH) provided the infrastructure for documentation, data management and data analysis. The Committee on Emergency Medicine, Intensive Care and Trauma Management (Sektion NIS) of the German Trauma Society provides scientific leadership. Pseudonymized data of participating hospitals are submit to a central database via web-based application. Most of the participating hospitals are located in Germany, but an increased number of hospitals in other countries contribute data as well. Currently, approximately 30,000 cases from more than 650 hospitals are entered into the database per year.

### TraumaNetzwerk DGU®

TraumaNetzwerk DGU® aims to improve the quality and reliability of care for severely injured individuals nationwide.

Trauma networks are formed to guarantee high-quality medical care for multiple trauma patients.

The requirements for a trauma network are the existence of one supra-regional trauma center, 2 regional trauma centers and 3 local trauma centers and the implementation of guidelines for trauma patients with special injuries such as spinal cord injuries or burns. The Whitebook Medical Care of the Severely Injured [18] provides detailed information about the functions of the supra-regional, regional and local trauma centers within a trauma network. The equipment, staff and facilities are defined. The centers are classified in the TraumaNetzwerk DGU® according to the level of care (level I, II and III) within the German health care system [19].

The present study is in line with the publication guidelines of the TraumaRegister DGU® and was registered as TR-DGU project ID 2018–001 N. The Ethics Committee Kiel, Schleswig-Holstein, examined and approved the study (AZ D527/18).

### Patients

A total of 58,939 trauma patients were evaluated from 2007 to 2017, and the data were analyzed retrospectively. Only data documented in the TraumaRegister DGU® were analyzed.

Not all patient data were available for all analyses. If the data were incomplete for a given evaluation, that patient was excluded from that analysis. The worst injury was considered an Abbreviated Injury Scale (AIS) score of 3 or higher (MAIS 3+). All data were taken from TraumaRegister DGU®. All patients between 1 and 100 years old with a primary admission and documented time of admission were included. Secondary admissions were excluded. We included all participating hospitals within Germany. Approximately 585 hospitals were included.

### Time periods

A day was divided into four time periods: 6.00 am–11.59 am (morning) (*n* = 14,496 patients), 12.00 pm–5.59 pm (afternoon) (*n* = 24,226 patients), 6.00 pm–11.59 pm (evening) (*n* = 16,278 patients), and 0.00 am–5.59 am (night) (*n* = 3939 patients). Trauma patients were grouped according to their time of admission. Patients who arrived at the hospital between 6.00 and 11.59 am composed the morning group. Trauma patients with an arrival time of 12.00 pm were added to the afternoon group. In the following analyses, we differentiated between day (6.00 am- 11.59 am, 12.00 pm- 5.59 pm) and night (6.00 pm–11.59 pm, 0.00 am–5.59 am).

### Statistical analysis

Statistical analyses were performed with SPSS 24.0. (IBM, Armonk, NY, USA) and GraphPad Prism 7 (GraphPad Software, Inc., USA). For descriptive analyses, the results are presented as the means ± standard deviations (SDs). For comparison of means ± standard deviations, we used one-way ANOVA. Differences in the proportions between groups were tested using the chi-squared test. To compare results across different time groups, we used the Kruskal-Wallis test. The unpaired t-test with Welch’s correction was used to calculate the mean values and SDs. The data are presented as the means and SDs for continuous measurements and as numbers (percentages) for categorical variables. Statistical significance was defined as *p* < 0.05. The large sample size resulted in very small *p*-values; thus, the *p*-values should be interpreted with caution. In addition to statistical significance, the clinical relevance of the observed differences always needs to be considered.

A logistic regression analysis of the outcomes in multiple trauma patients was used to identify the influencing factors. The important variables included the RISC II score (a combination of 13 different factors) [20], trauma center level, time of day, year of trauma, and the performance of whole-body multislice CT. The RISC II score was chosen as a predictor of mortality because it had a significantly larger area under the ROC curve (AUROC) than the other predictors [20]. In the development data set, the discrimination of the RISC II score, as reflected in the AUROC, was 0.947 (0.944–0.951) when all available data were included [20]. The AUROC in this study was: 0.939 (95% CI 0.936–0.941).

The regression coefficient B was determined as a statistical measure of the average functional relationship between the dependent and independent variables. The standard error represents the average distance that the observed values fall from the regression line, and Exp (B) is the exponentiation of the B coefficient, which is an odds ratio. Odds ratios are presented with their respective 95% confidence intervals.

## Results

A total of 58,939 patients were identified from 2007 to 2017, of whom 14,496 arrived from 6.00 am–11.59 am (24.6%), 24,226 arrived from 12.00 pm–5.59 pm (41.1%), 16,278 arrived from 6.00 pm–11.59 pm (27.6%) and 3939 arrived from 0.00 am–5.59 am (6.7%).

### Clinical characteristics in different time periods

Different clinical parameters of the trauma patients were evaluated to identify substantial differences between patients admitted during the day and those admitted at night (Table 1). The most common causes of injury in patients during all admission periods were car accidents and low falls (< 3 m) (Table 1). Among patients admitted at night, the percentages admitted due to car accidents and low falls were the highest. Motorcycle and pedestrian accidents accounted for the greatest percentages of admissions between 6.00 pm and 11.59 pm (*p* < 0.0001).

**Table 1:** Clinical characteristics of multiple trauma patients in different time periods (6.00 am- 11.59 am, 12.00 pm- 5.59 pm, 6.00 pm–11.59 pm, 0.00 am–5.59 am). *P* values are shown for all data

	6.00 am–11.59 am (morning)	12.00 pm–05.59 pm (afternoon)	6.00 pm–11.59 pm (evening)	0.00 am–5.59 am (night)	total	
All multiple trauma patients	14,496	24,226	16,278	3939	58,939	
Sex						
Male	9780 (67.5%)	17,059 (70.4%)	11,389 (70.0%)	2786 (70.7%)	41,014	
female	4716 (32.5%)	7167 (29.6%)	4889 (30.0%)	1153 (29.3%)	17,925	< 0,0001
Cause of trauma						
Car	3203 (22.6%)	4791 (20.2%)	3219 (20.2%)	970 (25.3%)	12,183	
Motorcycle	1218 (8.6%)	2820 (11.9%)	2512 (15.8%)	159 (4.1%)	6709	
Bicycle	1326 (9.3%)	2237 (9.4%)	1466 (9.2%)	126 (3.3%)	5155	
Pedestrian	1147 (8.1%)	1637 (6.9%)	1499 (9.4%)	159 (4.1%)	4442	
Fall > 3 m	2412 (17%)	4516 (19.0%)	2045 (12.8%)	754 (19.6%)	9727	
Fall< 3 m	3371 (23.8%)	5342 (22.5%)	3637 (22.8%)	1163 (30.3%)	13,513	
Others	1510 (10.6%)	2375 (10%)	1556 (9.8%)	508 (13.2%)	5949	< 0,0001
Age mean	54.6	55.3	50.1	49.42	52.36	< 0,0001
standard deviation	21.5	21.6	22.6	22.8	22.13	
n	14,474	24,197	16,255	3932	58,858	
ISS mean	21.0	20.6	21.0	21.4	21.0	< 0,0001
standard deviation	11.7	11.4	11.5	11.5	11.5	
n	14,496	24,226	16,278	3939	58,939	
GCS preclinical mean	12.4	12.6	12.0	11.6	12.15	< 0,0001
standard deviation	4.1	4.0	4.3	4.4	4.2	
n	13,582	22,760	15,302	3686	55,330	
AIS ≥3 head	5819 (40.1%)	9199 (38.0%)	6974 (42.8%)	1848 (46.9%)	23,840	< 0,0001
AIS ≥3 thorax	7052 (48.6%)	12,317 (50.8%)	7786 (47.8%)	1794 (45.5%)	28,949	< 0,0001
AIS ≥3 abdomen	1807 (12.5%)	2685 (11.1%)	1864 (11.5%)	502 (12.7%)	6858	< 0,0001
Trauma care						
Level I trauma center	8117 (56.0%)	14,344 (59.2%)	9847 (60.5%)	2365 (60.0%)	34,673	
Level II trauma center	4781 (33.0%)	7453 (30.8%)	4963 (30.5%)	1208 (30.7%)	18,405	
Level III trauma center	1598 (11.0%)	2429 (10.0%)	1468 (9.0%)	366 (9.3%)	5861	< 0,0001
mortality	2075 (14.3%)	3121 (12.9%)	2217 (13.6%)	619 (15.7%)	8032	< 0,0001

The percentage of male trauma patients was higher than the percentage of female trauma patients in all evaluated time periods and days.

Severe trauma patients admitted at night were significantly younger than those admitted during the day (*p* < 0.0001) (Table 1). Among the patients admitted at night, the mean age was 49.4 ± 22.8 years (*p* < 0.0001).

Blunt trauma was reported for the majority of trauma patients (78,748 patients). A higher percentage of patients with penetrating trauma were admitted at night than during the day (276 patients, 7.4%, *n* = 3752).

The mean injury severity score (ISS) was higher in patients admitted in the evening (21.0 ± 11.6) and at night (21.4 ± 11.5) (Table 1) than in those admitted at other times.

The lowest mean Glasgow coma scale (GCS) scores were identified in the patients admitted in the evening (12.0 ± 4.3) and at night (11.5 ± 4.4). Patients admitted at night had the highest percentage with an AIS score ≥ 3 for head injuries (Table 1).

The highest proportion of patients with an AIS score ≥ 3 for thoracic injuries was identified in those admitted in the afternoon, with 12,317 patients (50.8%) (Table 1). The highest proportions of patients with an AIS score ≥ 3 for abdominal injuries were identified in those admitted at night, with 502 patients (12.7%), and in the morning, with 1807 patients (12.5%) (Table 1).

We evaluated the percentages of trauma patients who were treated in level I, level II and level III trauma centers. The majority of the patients [51,495 patients (59.6%)] were admitted to level I trauma centers. A total of 26,576 patients were admitted to level II centers (30.7%), and 8369 patients (9.7%) were admitted to level III trauma centers.

### Time intervals

Time plays an important role in multiple trauma management (Table 2). A longer out-of-hospital time was found for patients admitted during the evening and at night (63.8 min, 65.5 min) than for those admitted during the day (Table 2). Interestingly, the longest waiting time in the emergency room occurred in the morning and not at night.

**Table 2:** Time intervals to the initiation of multiple trauma care in different time periods (6.00 am- 11.59 am, 12.00 pm- 5.59 pm, 6.00 pm–11.59 pm, 0.00 am–5.59 am). *P* values are shown for all data

		6.00 am–11.59 am (morning)	12.00 pm- 5.59 pm afternoon)	6 pm- 11.59 pm (evening)	0.00 am- 5.59 (night)	total	
Out-of-hospital Minutes	Mean value	61.4	62.0	63.8	65.5	63.2	< 0,0001
	Standard deviation	27.9	27.1	28.6	31.5	28.8	
	n	11,591	19,224	12,781	3189	46,785	
Time at the trauma roomminutes	Mean value	62.2	60.6	60.8	60.8	61.1	< 0,0001
	Standard deviation	43.4	40.7	39.4	36.3	40.0	
	n	4098	6923	4736	1151	16,908	
Time from arrival at the trauma room until X-ray minutes	Mean value	16.8	15.4	14.7	16.2	15.8	< 0,0001
	Standard deviation	21.3	19.7	18.6	19.8	19.9	
	n	5346	8678	5630	1378	21,032	
Time from arrival at the trauma room until CT minutes	Mean value	23.7	22.5	22.6	24.3	23.3	< 0,0001
	Standard deviation	19.3	17.8	17.4	18.1	18.2	
	n	12,386	20,888	14,172	3443	50,889	
Time from trauma room emergency operation minutes	Mean value	73.3	73.3	74.7	72.9	73.55	< 0,0001
	Standard deviation	27.6	27.1	27.4	25.8	27.0	
	n	693	1215	933	225	3066	
Days at ICU	Mean value	6.7	6.8	7.1	7.1	6.9	< 0,0001
	Standard deviation	10.6	10.7	10.9	10.9	10.8	
	n	14,496	24,226	16,278	3939	58,939	
Days at hospital	Mean value	17.6	18.2	18.1	16.8	17.7	< 0,0001
	Standard deviation	17.5	18.5	17.9	17.3	17.8	
	n	14,495	24,223	16,276	3939	58,933	

The longest interval from arrival at the emergency room to the performance of a CT scan occurred at night (Table 2). The time between arrival and the performance of an X-ray was also evaluated, and the longest intervals occurred in the morning (16.8 min) and at night (16.2 min) (Table 2). The length of hospital stay and days in the ICU showed slight variations (Table 2).

### Differences among levels 1, 2 and 3 hospitals

We also evaluated differences among trauma centers of varying levels during different time periods. The majority of multiple trauma patients were treated in level I hospitals (51,495 patients, 59.6%). A total of 26,576 patients (30.7%) were examined in level II centers, and 8369 patients (9.7%) were examined in level III centers. The majority of patients were treated in level I trauma centers independent of the time of the day.

The patients treated at the three levels of trauma centers had significant differences in injury severity, with the highest mean ISS identified in patients treated at level I centers and the lowest ISS identified in patients treated at level III trauma centers (Table 3). Unlike among patients treated at level II and III trauma centers, those treated at level I trauma centers did not differ significantly in their ISS based on whether they were treated during the day or at night.

**Table 3:** Differences among level 1, 2 and 3 trauma centers in different time periods

			6.00 am–11.59 am morning)	12.00 pm- 5.59 pm (afternoon)	6 pm- 11.59 pm (evening)	0.00 am- 5.59 am (night)	total	
**ISS**	Level I	Mean value	22.9	22.1	22.3	22.2	22.4	< 0,0001
		Standard deviation	12.6	12.0	12.0	11.6	12.1	
		n	8117	14,344	9847	2365	34,673	
	Level II	Mean value	19.4	19.1	19.6	20.8	19.7	< 0,0001
		Standard deviation	10.4	10.3	10.6	11.5	10.7	
		n	4781	7453	4963	1208	18,405	
	Level III	Mean value	16.3	16.7	17.0	18.5	17.1	< 0,0001
		Standard deviation	8.6	8.8	9.5	10.3	9.3	
		n	1598	2429	1468	366	5861	
**Time from arrival at the trauma room until CT scan minutes**	Level I	Mean value	21.0	20.3	20.7	21.9	21.0	< 0,0001
		Standard deviation	15.5	14.8	14.8	14.5	14.9	
		n	7324	12,974	8952	2130	31,380	
	Level II	Mean value	26.0	24.8	24.9	27.0	25.7	< 0,0001
		Standard deviation	21.3	19.9	19.3	20.4	20.2	
		n	4001	6298	4233	1057	15,589	
	Level III	Mean value	33.6	31.3	31.0	33.3	32.3	< 0,0001
		Standard deviation	28.0	25.4	25.5	28.3	26.8	
		n	1061	1616	987	256	3920	
**Time from trauma room until emergency operation minutes**	Level I	Mean value	72.9	73.0	74.8	72.8	73.4	< 0,0001
		Standard deviation	26.9	26.3	26.2	26.1	26.4	
		n	467	820	611	165	2063	
	Level II	Mean value	76.6	74.8	74.8	73.9	75.0	< 0,0001
		Standard deviation	27.2	27.6	28.5	24.7	27.0	
		n	180	323	269	50	822	
	Level III	Mean value	64.1	71.3	73.3	69.3	69.5	< 0,0001
		Standard deviation	34.6	32.9	35.6	28.0	32.8	
		n	46	72	53	10	181	

The time until imaging was performed was longer in level III trauma centers than in level I or II trauma centers at all times. Differences between the levels of trauma centers increased slightly at night.

We evaluated the times between hospital admission and the performance of a CT scan and admission and the performance of an emergency operation (Table 3). The time until the CT scan was longer in level III trauma centers than in level I and II trauma centers. The shortest time to the performance of a CT scan was found in level I trauma centers during all time periods. We found the longest time to imaging at night in all trauma centers (level I: 21.9 min, level II: 27.0 min, level III: 33.3 min). Emergency operations were defined as operations performed after or during the initial assessment due to life-threatening injuries. The time interval until imaging did not delay the time until emergency operation in different levels of trauma centers.

### RISC II scores and mortality

With regard to survival, we focused on the predicted (RISC II score) and observed mortality in different time intervals. The RISC II score and mortality were correlated in all time intervals. We found the highest mortality rate of 15.7% at night (95% confidence interval 14.6–16.9), when the predicted mortality was 14.5%. A relatively higher than predicted mortality rate was also observed in the morning (14.3, 95% confidence interval 13.7 and 14.9). The predicted mortality based on the RISC II score was 13.4% (Table 4). The mortality rate was significantly lower in the afternoon (12.9, 95% confidence interval 12.5–13.3, predicted mortality: 12.4%) than at night (15.7%; 95% confidence interval 14.6–16.9, predicted mortality: 14.5%).

**Table 4:** RISC II scores and mortality rates in multiple trauma patients in different time periods. SMR: standardized mortality ratio; RISCII: Revised Injury Severity Classification II

	6.00 am–11.59 am (morning)	12.00 pm- 5.59 pm (afternoon)	6.00 pm- 11.59 pm (evening)	0.00 am- 5.59 am (night)
**total**	14,496	24,226	16,278	3939
**‘mortality**	14.3	12.9	13.6	15.7
**95%** **confidence interval** **Low value-High value**	13.7–14.9	12.5–13.3	13.1–14.1	14.6–16.9
**RISC II**	13.4	12.4	13.0	14.5
**SMR**	1.07	1.04	1.05	1.08
**95%** **confidence interval** **Low value-High value**	1.0–1.1	1.0–1.1	1.0–1.1	1.0–1.2

### Regression analysis

A logistic regression analysis of mortality was performed to identify the factors influencing the mortality rate in multiple trauma patients. The odds ratio and regression coefficient varied during different time periods within a day. The RISC II score, performance of a CT scan and level of trauma center were known predictors. The year was adjusted for in the model. An effect of the time of arrival on the mortality rate was not observed (Table 5).

**Table 5:** Logistic regression analysis of the association of mortality in multiple trauma patients with different predictors: RISC II score, level of trauma center, time period, year, and performance of multislice CT. df: degrees of freedom, sig.: significance, exp. (B): exponentiation of the B coefficient

	regression coefficients	wald	significance	Exp(B)	95% confidence interval Low value	High value
**RISC II**	0.945	10,606.2981	0.000	0.389	0.382	0.396
**level**		1.256	0.534			
**Level I**	0.016	0.187	0.666	1.016	0.947	1.090
**Level II**	0.061	0.881	0.348	0.940	0.827	1.069
**Time period**		9.422	0.051			
**6.00 am–11.59 am (morning)**	0.112	5.691	0.017	1.118	1.020	1.226
**12.00 pm–05.59 pm (afternoon)**	0.075	3.317	0.069	1.078	0.994	1.168
**6.00 pm–23.59 pm (evening)**	0.112	6.161	0.013	1.119	1.024	1.222
**0.00 am–5.59 am (night)**	0.124	2.859	0.091	1.132	0.980	1.307
**Year**		7.175	0.127			
**2014**	0.017	0.118	0.732	1.017	0.922	1.122
**2015**	0.033	0.450	0.502	1.034	0.938	1.138
**2016**	0.083	2.946	0.086	0.920	0.837	1.012
**2017**	0.037	0.616	0.433	0.963	0.877	1.058
**Multislice Whole-body CT**	0.210	29.592	0.000	0.811	0.752	0.874
**constant**	0.141	6.892	0.009	1.152		

## Discussion

The present study compared different parameters of multiple trauma care among various times of the day. Contrary to the findings in previous studies, we showed that the time of admission had no effect on the length of hospital stay or outcome among multiple trauma patients. We believe that this is evidence that a consistent level of trauma care is available 24 h per day.

Unlike in previous evaluations [21], the majority of multiple trauma patients were male, irrespective of the time of day they were admitted. Most injuries occur in environments and circumstances that involve sex-based differences in behavior; hence, there is a higher prevalence of all injury mechanisms in males, especially traffic accidents [22].

The age of multiple trauma patients differed significantly between those admitted during the day and at night. Trauma patients admitted at night were significantly younger than those admitted during the day, which agrees with previous findings [23].

During the day, many traffic accidents are caused by commuter traffic [24]. The group of multiple trauma patients older than 65 years has increased significantly in recent decades, although older people tend to go out during the day more than they do at night [24]. Traffic accidents at night might be caused by people traveling to various party venues [4]. In particular, alcohol-related crashes involving young drivers are relatively more common at night [25].

Traffic accidents are the most common cause of multiple trauma [26]. Most car and motorcycle accidents occur in the evening. In Germany, approximately 80% of traumatic injuries are caused by motor vehicle accidents [27]. At noon and during the afternoon rush hour, there are relatively more traffic accidents [4].

Minor falls are a major cause of accidents. Especially in the elderly population, minor falls are a common cause of multiple trauma, and the number of falls has increased in recent years [28, 29]. Predisposing risk factors that are becoming increasingly important due to the increase in the population of older patients might be comorbidities, age-associated loss of strength or balance and the use of certain medications [30].

The percentage of suspected suicides was the highest at night. Several studies have found a circadian variation in suicide rates depending on age, with a morning peak for older patients and an evening peak for younger patients [31]. Biologic factors, such as sunshine and daylight cycles, as well as biomarkers, such as melatonin, serotonin and cortisol, play major roles in circadian variations in suicide [31].

Our study did not identify any substantial difference in the ISS and GCS scores based on the time of day. The highest mean ISS and the lowest preclinical GCS score occurred at night. The percentages of patients with head injuries and penetrating trauma peaked at night. Dim lighting contributes to the severity of traffic injuries [32]. Reaction times are substantially longer under poor conditions [32]. Factors such as fatigue and alcohol contribute to the impaired ability of drivers to avoid severe collisions at night [32].

Time is critically important in multiple trauma patients [32]. Several authors have shown a correlation between the time to initial trauma care and the long-term outcome [33]. The outcomes differ significantly between emergency patients admitted during the day and at night, and the mortality rate is higher among those admitted at night [34]. Cognitive performance and hand-eye coordination are impaired in professionals working at night [35]. An analysis of the quantity and quality of emergency physicians has shown that physicians working night were slower at intubation and made more errors [36].

In our evaluation, the prehospital period was longer at night. Emergency treatment, especially in cases of vehicle accidents and those requiring technical rescue, can be complicated and time-consuming in the dark. In addition, rescue stations might not be as well staffed as they are during the day.

Multiple severe injuries necessitate more stabilizing interventions and prolong the out-of-hospital time [37]. There is a general consensus that critical and life-threatening injuries must be managed during the out-of-hospital phase even when this causes delay [37].

During the night shift, the time spent waiting in the emergency room and time from arrival at the emergency room until the performance of an emergency operation were short and comparable with the times during the day. These findings are contrary to previous results since most hospitals are understaffed at night, and the performance of physicians is worse at night than during the day [15].

Egol et al. suggested that understaffed hospitals and overfatigued physicians were responsible for the higher mortality at night [38]. A higher risk of mortality at night was also observed for patients admitted to level III and IV hospitals in the United States [38]. We found longer time periods until the performance of CT scans in level III trauma independent of the time of day, but delay did not cause a subsequent delay in the performance of emergency operations or a prolonged hospital stay.

Multiple trauma management in the emergency room seems paradoxical, with fast treatment considered essential, while the precise diagnostic imaging needed is time consuming [39].

Parsch et al. [40] evaluated the times to different medical interventions, such as intubation, venous catheter insertion and splinting, during business and nonbusiness hours. There were no differences in the trauma room treatment parameters between the day and night shifts [40]. It may be that the knowledge and experience gained during previous evaluations ensures a consistent level of trauma care even at night. Patients with high ISSs and severe head injuries were found to have longer stays in the emergency department than trauma patients with lower ISSs [41], but we did not find relevant differences in ISSs in level I trauma centers. Higher ISSs were observed for level III trauma centers at night, but higher ISSs did not correlate with a longer duration of care.

One explanation might be that the implementation of the European Working Time Directive has changed the working conditions in hospitals over the past years [42–44]. The primary aim of the law regarding working hours was to protect clinical employees from being made to work overtime for the benefit of patients [45]. Reducing the amount of time spent working, performing shift work and increasing rest time might have helped improve the quality of patient care and accelerated the time to the performance of procedures in all clinical departments at night.

We did not find fundamental differences in the length of stay in the ICU or length of stay in the hospital based on the time of arrival, which correlates with the findings of Morales et al. [46].

It is generally accepted that the early identification of injuries with rapid resuscitation and management can improve the survival of multiple trauma patients [41]. We evaluated the RISC II score during the day and at night. The RISC II score is a prognostic factor for the outcome, independent of arrival time and level of trauma center [20]. The highest RISC II score occurred in the patients admitted at night, which correlated with an increase in severe head injuries and a lower GCS in our evaluation. Increasing injury severity results in higher RISC II scores [20].

We found no essential difference between the RISC II score and mortality rate in any time period, which highlights the high prognostic relevance of the RISC II score. There were no differences between the expected and observed mortality rates. The highest RISC II score and highest mortality rate were observed at night. The RISC II score combines different factors that are predictive of the survival of trauma patients [20].

The specifics of the trauma center and arrival time are not included in this score; therefore, a regression analysis was performed. Our regression analysis showed no correlation between the time of day and mortality rate. Time of arrival and trauma care did not affect the mortality rate. Barbosa et al. evaluated trauma patients who underwent surgery and found that admission at night was an independent predictor of hospital mortality [47], but there was no focus on multiple trauma patients, and only patients from one public trauma center were included.

Hirose et al. divided a day into two intervals and showed a negative impact of admission at night on the outcome in emergency trauma patients in Japan [15]. The emergency medical services and medical system in Japan differ from those in Europe or the United States, and personnel are on duty for a full 24-h period [15]. Even when materials and medical staff are limited, the optimal provision of trauma care remains critical. Laupland et al. did not find a difference between patients who arrived at night and those who arrived during the day, which may reflect the highly developed trauma care system in Canada [48]. There seems to be a high degree of variation in trauma care, and what holds true in one region is not necessarily applicable in another.

We highlight that the admission of multiple trauma patients at night does not have a negative effect on outcomes or mortality in German trauma centers. These findings reflect the quality of multiple trauma care in German hospitals around the clock. We believe that understanding the difficulties and weaknesses in emergency management at night has fundamentally improved trauma care at night.

## Conclusion

Multiple trauma patients admitted at night were younger than those admitted during the day, and the mean ISS was higher in patients admitted at night than in those admitted during the day. Despite the fact that there were differences in injuries and injury severity, the examined time intervals for trauma care differed slightly, without a clinical impact. Patients admitted during night had higher RISC II scores and a higher mortality rate, but admission at night was not a predictor of higher mortality. In German hospitals, a consistent level of multiple trauma care is provided 24 h per day.

## Abbreviations

AIS: Abbreviated Injury Scale
AUC: Academy for Trauma Surgery
am: Ante meridiem
CT: Computer-assisted Tomography
df: Degrees of freedom
exp.: Exponentiation of the B coefficient
GCS: Glasgow Coma Scale
ICU: Intensive Care Unit
ICM: Intermediate Care Medicine
ISS: Injury Severity Score
min: Minutes
n: Number of
pm: Post meridiem
RISC II: Revised Injury Severity Classification II
Sektion NIS: Committee on Emergency Medicine, Intensive Care and Trauma Management
SD: Standard Deviations
sig.: Significance
TraumaRegister DGU®: TraumaRegister der Deutschen Gesellschaft für Unfallchirurgie
TR-DGU: TraumaRegister der Deutschen Gesellschaft für Unfallchirurgie
TNW: TraumaNetzwerk DGU®
